# Invasive infections due to *Magnusiomyces capitatus*: case report and review of its prevalence in China

**DOI:** 10.1080/21501203.2021.2000059

**Published:** 2021-11-22

**Authors:** Mingxun Zhu, Liang Yan, Sybren de Hoog, Wanqing Liao, Hong Zhang, Rongfen Zhao, Shuwen Deng

**Affiliations:** a Department of Medical Microbiology, The People’s Hospital of Suzhou New District, Suzhou, Jiangsu, China; b Department of Dermatology, The Pla General Hospital of Central Theater Command, Wuhan, China; c Shanghai Key Laboratory of Medical Molecular Mycology, Changzheng Hospital, Second Military Medical University, Shanghai, China; d Centre of Expertise in Mycology of Radboud University Medical Centre/Canisius Wilhelmina Hospital, Nijmegen, The Netherlands

**Keywords:** *Magnusiomyces capitatus*, *Saprochaete capitata*/*Geotrichum capitatum*, fungemia, invasive fungal infection, China

## Abstract

*Magnusiomyces capitatus* is an emerging opportunistic yeast, thus far mainly reported from the Western world where fungemia is the most frequent presentation in immunocompromised patients with high mortality. We described a rare case of *Magnusiomyces capitatus* infection from our hospital in China and reviewed six further cases published to date in Chinese literature. It is noted that half more of the cases (4/7) presented with fungemia in younger, immunosuppressed patients, whereas the remaining cases were with pneumonia in elderly, immunocompetent patients. All seven Chinese cases had favourable outcome with antifungal therapy. Based on the limited in vitro and clinical data, a combination of amphotericin B either with 5-fluorocytosine or voriconazole for fungemia in immunocompromised patients, and although fluconazole is not recommended as first-line therapy in the guideline, in our study, fluconazole alone or with 5-fluorocytosine for local pulmonary infection in immunocompetent patients is effective with good outcome.

## Introduction

*Magnusiomyces* is a genus of arthroconidial yeasts in the order *Saccharomycetales*, family *Dipodascaceae,* at large phylogenetic distance from ascomycetous yeasts producing budding cells. Species of the genus was previously known under the names *Geotrichum/Dipodascus* (for anamorph and teleomorph, respectively), but after abandoning dual nomenclature for fungi, *Magnusiomyces* was chosen for filamentous yeasts producing bipodal asci containing ascospores enveloped by gelatinous sheaths (Kaplan E et al. 2017). The genus currently contains 14 species. The most important clinical species is *Magnusiomyces capitatus*; frequently used, obsolete names for this fungus applied in older medical literature are *Trichosporon* and *Blastoschizomyces*, in addition to *Geotrichum* (De Hoog and Smith 2004).

*M. capitatus* is an opportunistic pathogen with an underestimated frequency. Systemic infections have been reported as fungemia, endocarditis, and particularly pulmonary infections, prevalently occurring in patients with haematological disorders, and the mortality rate of disseminated infections is quite high regardless of antifungal treatment (Mazzocato et al. 2015). Infections are well documented in European countries such as Italy, Spain, and France (Mazzocato et al. 2015). However, Tanabe et al. reported that this species could be an infectious risk for immunocompetent patients (Tanabe and Patel 2018). An environmental niche of the fungus is in household dishwashers (Zalar et al. 2011; Döğen et al. 2013; Gümral et al. 2016), demonstrating its thermophilic nature and suggesting a possible source of contamination. Despite increasing attention for this fungus, only a relatively few case reports have been published from Asia (Ersoz et al. 2004; Subramanya Supram et al. 2015). Information from China is very limited as the few reported cases are written in Chinese language (Ersoz G et al. 2004; CLSI 2008; De Hoog et al. 2014; Subramanya Supram et al. 2015; Gao et al. 2015; Qian et al. 2016). Here, we describe a case from our hospital in Suzhou and review six further cases of *M. capitatus* infection published to date in the Chinese literature.

## Case presentation

A 74-year-old male with right upper lobe lobectomy due to squamous cell lung carcinoma 6 years ago, and a history of recurrent lower respiratory tract infections for the past 4 years, was admitted to our hospital. He had fever and a cough with sputum, and his white cells were 10.37 * 10^9^/L. His chest X-ray showed collapse of the lung at the right side. Based on clinical and radiological features, his disorder was diagnosed as pneumonia. Microbiological tests performed were all negative except that sputum yielded *M. capitatus* (KOH examination showed fungal hyphae consistently and positive cultures were obtained on several occasions). Unfortunately, bronchial alveolar lavage could not be performed due to the patient’s refusal. The patient improved after a therapy of fluconazole 400 mg per day for 7 weeks. However, the patient died of acute airway obstruction 1 year later although *M. capitatus* had been cleared from his sputum.

The fungus showed relatively expanding, cream coloured, dry, wrinkled colonies on SGA（sabouraud dextrose agar). Hyphae fragmented into arthroconidia. The effect of temperature on growth showed that the isolate was able to grow at 30°C, 37°C, and 40°C, but not 45°C. It was identified as *M. capitatus* morphologically and confirmed by sequences of rDNA ITS, D1/D2 of LSU, and partial *Rpb*2. The sequences were deposited in the GenBank database with accession numbers MK000896 (LSU), MH999810 (ITS), and MK585525 (*Rpb2*). The culture of the isolate was deposited in the reference collection of Westerdijk Institute with the accession number CBS15870.

In vitro antifungal susceptibility of the *M. capitatus* isolate was tested against 5-fluorocytosine, fluconazole, itraconazole, posaconazole, ravuconazole, caspofungin, and amphotericin B (all from Sigma Poole, United Kingdom), anidulafungin, voriconazole, and isavuconazole (Toronto Research Chemicals, Toronto, Ontario Canada), micafungin (Astellas Pharma Inc., TYO: 4503) according to the CLSI M27-A4 (CLSI 2008), with *Candida parapsilosis* ATCC22019 and *Candida krusei* ATCC6258 as quality control strains. The results showed that 5-fluorocytosine is the most active drug against *M*. *capitatus* in vitro with the lowest MIC (minimum inhibitory concentration) (0.125 μg/mL), followed by itraconazole and voriconazole (0.5 μg/mL, respectively), isavuconazole, ravuconazole, and micafungin (1 μg/mL, respectively), amphotericin B, posaconazole, and anidulafungin (2 μg/mL, respectively), fluconazole (4 μg/mL), and caspofungin (8 μg/mL) (Table 2).

**Table 1.** Summary of the clinical and microbiological profile of the seven Chinese cases

Parameter	Case 1	Case 2	Case 3	Case 4	Case 5	This case
Year	2011	2015	2018	2013	2018	2018
Age/gender	53/female	25/male	14/female	64/Male	92/male	74/male
Underlying disease	Lymphoma	Leukaemia	Aplastic anaemia	COPD, coronary heart disease	Diabetes, aged	Right upper lobe lobectomy
Chemotherapy	Yes	Yes	Yes	no	No	No
Clinical symptoms	Fever	Fever	Fever	cough	Cough, fever	Cough
Culture	Positive (blood)	Positive (blood)	Positive (blood)	positive (sputum, both KOH and culture)	Positive (sputum, both KOH and culture)	Positive (sputum, both KOH and culture)
Diagnosis	Fungemia	Fungemia	Fungemia	pneumonia	Pneumonia	Bronchiectasis and pneumonia
Pathogen identification	Sequencing, morphology	Morphology	Sequencing, morphology	morphology, biochemical methods*	Sequencing, morphology	Sequencing, morphology
G-test/GM	N	G/GM positive	GM positive	N	N	N
Therapy in vivo	AmB + 5-FC	Am B + Vor	AmB + Cas	Flu	Flu + 5-FC	Flu
outcome	Good	Good	Good	good	Good	good
reference	8	9	10	11	12	

* ATB Expression bacteria identification Instrument (French bio-Merieux).

AmB: amphotericin B; 5-FC: 5-fluorocytosine; Vor: voriconazole; Cas: caspofungin; Flu: fluconazole; N: no test.

**Table 2.** Results of in vitro antifungal susceptibility, clinical therapy, and outcome for the seven Chinese cases

Case	Method	*In vitro* antifungal susceptibility									Therapy in vivo	Response
		AmB	5-FC	FLU	ITR	VOR	POS	CAS	MICA	ANI		
1	Unknown	S	S	S	S	S	-	-	-	-	Amphotericin B + 5-fluorocytosine	Good
2	Unknown	S	S	I	I	S	-	-	-	-	Amphotericin B + voriconazole	Good
3	Unknown	S	S	-	-	S	-	-	-	-	Amphotericin B + caspofungin	Good
4	Agar diffusion	S	S	I	I	-	-	-	-	-	Amphotericin B + voriconazole	Good
5	ATB fungus	S	S	S	S	S	-	-	-	-	Fluconazole	Good
6	Agar diffusion	-	S	S	S	S	-	-	-	-	Fluconazole + 5-fluorocytosine	Good
This	M27-A (µg/mL)	2 (S)	0.125 (S)	4 (S)	0.5 (S)	0.5 (S)	2 (*)	8 (*)	1 (*)	2 (*)	Fluconazole	Good

*There is no interpretive breakpoint.

AmB: amphotericin B; 5-FC: 5-fluorocytosine; FLU: fluconazole; ITR: itraconazole; VOR: voriconazole; POS: posaconazole; CAS: caspofungin; MICA: micafungin; ANI: anidulafungin; S: susceptible; -: not test; I: intermediate.

Astellas Pharma

) according to the CLSI M27-A4 (CLSI 2008), with *Candida parapsilosis* ATCC22019 and *Candida krusei* ATCC6258 as quality control strains. The results showed that 5-fluorocytosine is the most active drug against *M*. *capitatus* in vitro with the lowest MIC (0.125 μg/mL), followed by itraconazole and voriconazole (0.5 μg/mL, respectively), isavuconazole, ravuconazole, and micafungin (1 μg/mL, respectively), amphotericin B, posaconazole, and anidulafungin (2 μg/mL, respectively), fluconazole (4 μg/mL), and caspofungin (8 μg/mL) (Table 2).

## Discussion

Numerous cases of the infection by *M. capitatus* have been reported from Western countries (De Hoog et al. 2014), but the disorder seems to be rare in Asia. A search for reports in Chinese Wanfang database (www.wanfangdata.com.cn/index.html) and CNKI (China National Knowledge Internet) revealed six cases of *M. capitatus* infection in China between 2011 and 2019 (Table 1)(Ying et al. 2011; Wang et al. 2013; Gao et al. 2015; Li et al. 2018; Shan et al. 2018). Consequently, seven cases of *Magnusiomyces* infection are known to date from China.

Clinical and microbiological profiles of all seven Chinese cases are summarised in Table 1. The infections occurred in patients with a wide range of age (13–92 years old). The gender distribution (male:female = 5:2) is similar to that reported in other studies (Mazzocato et al. 2015). In contrast to most reports from Western countries, in which the most frequent presentation is fungemia in immunocompromised patients (Mazzocato et al. 2015), only four out of seven Chinese patients presented with fungemia. These patients were younger (13, 14, 25, and 53 years old) and profoundly immunosuppressed due to chemotherapy for their underlying haematological diseases. Whereas the three patients with infections confined to the lungs were all immunocompetent and elderly (64, 74, and 92 years old), having diabetes or COPD as underlying disease.

Molecular identification of *M. capitatus* can be made with about 96–99% confidence by sequencing the ribosomal ITS and D1/D2 LSU regions and by sequencing *Rpb*2 (Kaplan et al. 2017), as was done for the isolate in the current report. As additional confirmation, our isolate was unable to grow at 45°C. As an alternative diagnostic method, detection of serum galactomannan (GM) has been reported in cases of disseminated infection, with the observation that the measurement of GM antigen seems to provide more reliable results when applied to serum (Bonini et al. 2008). Of the six published cases from China, five were verified either by sequencing, by a positive GM test, or by mass-spectrometric analysis besides morphology and one case was diagnosed by biochemical methods using ATB Expression bacteria identification Instrument (French bio-Merieux) besides morphology (Table 1).

For targeted treatment, “Global guideline for the diagnosis and management of rare yeast infections: an initiative of the ECMM in cooperation with ISHAM and ASM” was just published recently (Chen et al. 2021). This guideline summarised the consensus recommendations with regards to the diagnostic and therapeutic options for patients with these rare yeast infections including the genera *Saprochaete* and *Magnusiomyces*. Recommendations are to use an amphotericin B formulation with or without flucytosine, or with voriconazole for initial therapy, on the basis of clinical data (Chen et al. 2021). The clinical therapy and outcome for seven Chinese cases are summarised in Table 2. Among the seven Chinese cases, amphotericin B is the main drug utilised in combination regimens (with voriconazole, 5-fluorocytosine, or caspofungin) for the four immunocompromised patients with fungemia which were in agreement with those in guideline (Chen et al. 2021), although one case had good outcome using amphotericin B combined with caspofungin, but the effect of caspofungin alone remained unclear. However, fluconazole was proved to be the most effective drug, either in monotherapy or in combination therapy, applied in three immunocompetent patients with pneumonia where *M. capitatus* was confined to the lungs. which is different from the guideline (Chen et al. 2021) that fluconazole is not recommended for the first-line antifungal therapy for patients with *Magnusiomyces* spp. infections. This could be explained by In vitro antifungal results with low MIC to fluconazole for those isolates of *M. capitatus* from immunocompetent patients; it seems that isolates from immunocompetent patients are sensitive to fluconazole as shown in Table 2. Of note, antifungal therapy was successful in all seven Chinese cases either immunocompromised patients or immunocompetent patients. This is different from most European reports, where cases involving immunocompromised patients were accompanied by a high mortality rate (Mazzocato et al. 2015).

In vitro antifungal assays conducted in previous studies indicate that *M. capitatus* is susceptible to amphotericin B, voriconazole, and 5-fluorocytosine, less susceptible to fluconazole (MICs between 2 and 32 µg/mL) (Mazzocato et al. 2015; Kaplan E et al. 2017; Chen et al. 2021), and resistant to echinocandins (Chen et al. 2021). Results of antifungal susceptibility on those seven isolates of *M. capitatus* (Table 2) (Ying et al. 2011; Wang et al. 2013; Gao et al. 2015; Li et al. 2018; Shan et al. 2018) including the isolate from this case report are concordant with previous studies (Kaplan et al. 2017).
